# Efficacy of Probiotic Treatment in Alcoholic Liver Disease: A Systematic Review of Animal Studies

**DOI:** 10.3390/nu18040608

**Published:** 2026-02-12

**Authors:** Konrad Sosnowski, Robert Kucharski, Adam Przybyłkowski

**Affiliations:** Department of Gastroenterology and Internal Medicine, Medical University of Warsaw Banacha 1A, 02-091 Warsaw, Poland; konrad.sosnowski@wum.edu.pl (K.S.);

**Keywords:** alcohol-associated liver disease, probiotics, gut–liver axis, gut microbiota, intestinal barrier, animal models, systematic review

## Abstract

Background/Objectives: Alcohol-associated liver disease (ALD) is a major cause of chronic liver injury, in which disruption of the gut–liver axis plays a key pathogenic role. Probiotics have been proposed as a potential therapeutic strategy to mitigate alcohol-induced liver damage; however, the preclinical evidence has not been systematically synthesised. This systematic review aimed to evaluate and summarise the hepatoprotective effects of probiotic supplementation in experimental animal models of ALD. Methods: The review protocol was pre-registered in PROSPERO (CRD420250653666) and followed PRISMA guidelines. A systematic search was conducted across PubMed, EMBASE and AGRICOLA databases using relevant keywords from inception to 30 April 2025. We included preclinical randomised controlled trials evaluating single-strain probiotic interventions against placebo or untreated controls in animal models of ALD. Risk of bias was assessed using SYRCLE’s tool, and the certainty of evidence for critical outcomes was evaluated using the GRADE framework. A narrative synthesis was performed, as a quantitative meta-analysis was precluded by incomplete numerical outcome reporting. Results: From initial 628 records, 36 studies were included in the final synthesis. Probiotic supplementation consistently attenuated alcohol-induced liver injury, as evidenced by marked reductions in serum ALT and AST levels and improved liver histology. Mechanistically, probiotics restored gut barrier integrity, reduced systemic endotoxemia, and suppressed pro-inflammatory pathways. Furthermore, probiotic treatment effectively counteracted alcohol-induced gut dysbiosis by increasing microbial diversity and restoring taxonomic balance, notably by reversing the alcohol-induced expansion of Proteobacteria. Despite these consistent directional effects, the overall certainty of evidence for the critical outcomes was rated as very low. Conclusions: Although the preclinical literature suggests hepatoprotective effects of probiotics in experimental ALD, these findings should be interpreted with caution due to the very low certainty of evidence. The observed benefits are limited by methodological shortcomings, indirectness inherent to animal models, and incomplete outcome reporting. This review provides a structured preclinical framework to inform the design of future translational studies and well-controlled clinical trials evaluating probiotics as potential adjunctive therapies in human ALD.

## 1. Introduction

Alcohol-associated liver disease (ALD) remains a major cause of chronic liver injury and mortality worldwide, accounting for a substantial portion of cirrhosis and liver-related deaths [1]. Globally, ALD affects approximately 3.02 million individuals, with an annual incidence of 462,690 new cases and 354,250 deaths, and between 2000 and 2021 the prevalence of ALD increased by 32.60%, underscoring the growing global burden of the disease [2]. Alcohol-related liver disease is the leading cause of cirrhosis in Europe, North America, and Asia, accounting for nearly 60% of diagnoses, and since the onset of the COVID-19 pandemic in 2020, ALD-related mortality has increased at an accelerated and concerning rate [3]. Despite extensive research, effective pharmacological therapies are limited, and management still relies primarily on abstinence and supportive care [4]. This therapeutic gap highlights the need to develop novel, mechanism-based interventions that can address the multifaceted nature of ALD pathogenesis.

Emerging evidence points to the gut–liver axis as a pivotal point in mediating alcohol-induced liver injury. Chronic ethanol consumption leads to dysbiosis, reduces the microbial diversity and promotes the expansion of pro-inflammatory, lipopolysaccharide (LPS)-producing taxa [5,6]. These alterations lead to compromise of intestinal barrier integrity, allowing translocation of bacterial endotoxins into the portal circulation [7]. The resulting endotoxemia triggers hepatic inflammation, oxidative stress, and lipid accumulation—key processes that drive the progression from simple steatosis to steatohepatitis and fibrosis [8].

Probiotics have been proposed as a promising therapeutic approach to counteract these effects [9]. By restoring microbial balance, enhancing tight-junction integrity, and modulating oxidative and inflammatory pathways, probiotics may interrupt the self-perpetuating cycle of dysbiosis, endotoxemia, and liver injury that characterises ALD [10]. To date, two systematic reviews with meta-analyses have been published—one of which appeared after completion of the present review—encompassing 9 and 12 clinical studies, respectively; however, the preclinical evidence derived from animal studies has not yet been systematically synthesised [11,12].

To address this gap, we conducted a systematic synthesis of preclinical data to comprehensively evaluate probiotic interventions in experimental models of alcohol-induced liver disease. Specifically, we aimed to (i) identify consistent effects of probiotics on key biochemical, histological, and molecular markers of hepatic injury; (ii) characterise their influence on the gut microbiota, intestinal barrier function, and systemic endotoxemia; and (iii) integrate these findings into a coherent mechanistic framework linking probiotic activity with protection against alcohol-induced liver injury.

## 2. Materials and Methods

### 2.1. Review Protocol

We pre-registered and published the design and methodology of this systematic review a priori in the PROSPERO database (registration ID: https://www.crd.york.ac.uk/PROSPERO/view/CRD420250653666; published on 3 March 2025). We conducted this review in accordance with the PRISMA guidelines and followed established principles for conducting systematic preclinical reviews.

### 2.2. Search Strategy

To identify all relevant experimental studies, we conducted a literature search across three electronic databases: PubMed, EMBASE, and AGRICOLA. We limited searches to English-language publications reporting randomised animal trials. We did not include any registers or grey literature sources. The prompt used for each database is published in the study protocol in the PROSPERO database and in Supplementary Materials S1.

### 2.3. Eligibility Criteria

We selected studies using the PICO framework:

- Population: Non-human, mammal animal models of alcoholic liver disease (ALD).
- Intervention: Any probiotic treatment consisting of a singular strain, irrespective of dose, or formulation.
- Comparator: Placebo or untreated control groups.
- Outcomes: Primary outcomes included histopathological indices of liver injury and serum biomarkers (ALT and AST activity). Secondary outcomes included measures of lipid profile, systemic inflammation, gut barrier integrity, and gut microbiota composition.

Only randomised experimental designs were eligible for inclusion.

We deliberately limited the scope of this review to single-strain probiotic interventions to reduce potential confounding arising from inter-strain interactions and heterogeneity in multi-strain formulations, thereby allowing a more precise interpretation of strain-specific effects.

### 2.4. Study Selection and Data Extraction

Two reviewers (K.S. and R.K.) independently screened titles and abstracts, followed by full-text assessment of potentially eligible studies. We resolved disagreements by discussion, with arbitration by a third reviewer (A.P.) when necessary. Two investigators (K.S. and R.K) independently extracted data using a standardised form, capturing study characteristics, animal model details, probiotic intervention parameters, and all predefined outcomes. The primary outcomes were histopathological features of liver injury and biomarkers of liver function, including liver histology scores and biochemical markers such as alanine aminotransferase, aspartate aminotransferase, alkaline phosphatase, bilirubin, and inflammatory cytokines. Secondary outcomes comprised changes in gut microbiota composition and gut–liver axis-related parameters, including intestinal permeability, endotoxemia, and systemic inflammation; data were extracted in their original formats and standardised when necessary for comparative analysis. In multi-arm studies, only the arms in which the probiotic was administered as stand-alone therapeutic option were included in the analysis. We tried to contact the corresponding authors for clarification when the data were incomplete or ambiguous.

### 2.5. Risk of Bias Assessment

We assessed methodological quality using SYRCLE’s risk of bias tool, which evaluates ten domains of bias adapted from the Cochrane framework [13]. Two reviewers (K.S. and R.K.) conducted independent assessments.

In addition, the certainty of evidence for each critical outcome was evaluated using the Grading of Recommendations, Assessment, Development and Evaluation (GRADE) framework. As the body of evidence consisted of randomised animal studies, the certainty of evidence was initially rated as high and subsequently downgraded based on five predefined domains: risk of bias, inconsistency, indirectness, imprecision, and publication bias. Judgments for the GRADE risk-of-bias domain were informed by the SYRCLE assessments and applied at the outcome level in accordance with GRADE guidance. Downgrading decisions were made when serious or very serious concerns were identified in any domain, and the final certainty of evidence was classified as high, moderate, low, or very low. This assessment focused primarily on critical outcomes, including biochemical markers of liver injury (serum ALT and AST) and histopathological indicators of liver damage.

### 2.6. Data Synthesis

A quantitative meta-analysis was not feasible due to substantial limitations in the availability and consistency of quantitative outcome data across the included studies. Numerical data required for quantitative synthesis were reported in only a small proportion of studies; for example, quantitative values for serum ALT and AST activity, including means and measures of variability, were available in only 5 of the 36 included studies. In the remaining studies, outcomes were reported descriptively or presented exclusively in graphical form without accompanying numerical values, precluding reliable extraction of effect sizes and measures of precision.

To address this limitation, we attempted to obtain missing numerical data by contacting the corresponding authors; however, these efforts did not yield sufficient additional information to permit quantitative pooling. Consequently, a structured narrative synthesis was undertaken.

## 3. Results

The complete dataset summarising all extracted variables and outcomes was too extensive to be presented within the main text and is therefore provided as Supplementary Materials S2.

### 3.1. PRISMA Workflow Chart

We conducted a comprehensive search across three electronic databases—PubMed (197 records), EMBASE (346 records), and AGRICOLA (85 records)—yielding a total of 628 records. No additional registers or grey literature sources were used.

Following title and abstract screening, we excluded 538 records. We sought to retrieve 99 articles, but 6 were unavailable (we tried to contact the corresponding authors for the text). We evaluated all the reports against the predefined inclusion and exclusion criteria. A total of 48 articles were excluded for the following reasons: duplicate records (*n* = 22); failure to meet eligibility criteria (*n* = 15); conference abstracts without available full text (*n* = 9); and non-English language publications (*n* = 2). Any discrepancies in study selection were resolved by the supervising reviewer.

Studies were excluded for the following reasons: use of multiple probiotic strains or compound/enriched probiotic formulations [14,15,16,17,18,19,20,21,22,23,24,25]; inclusion of additional experimental interventions in the alcohol-associated liver disease model (intraperitoneal lipopolysaccharide administration) [20]; use of genetically modified bacterial strains [26]; or non-mammalian study models [27]. One additional study was excluded after full-text review because it investigated *Porphyromonas gingivalis* as a disease-exacerbating factor rather than evaluating a probiotic intervention [28].

**Figure 1:**
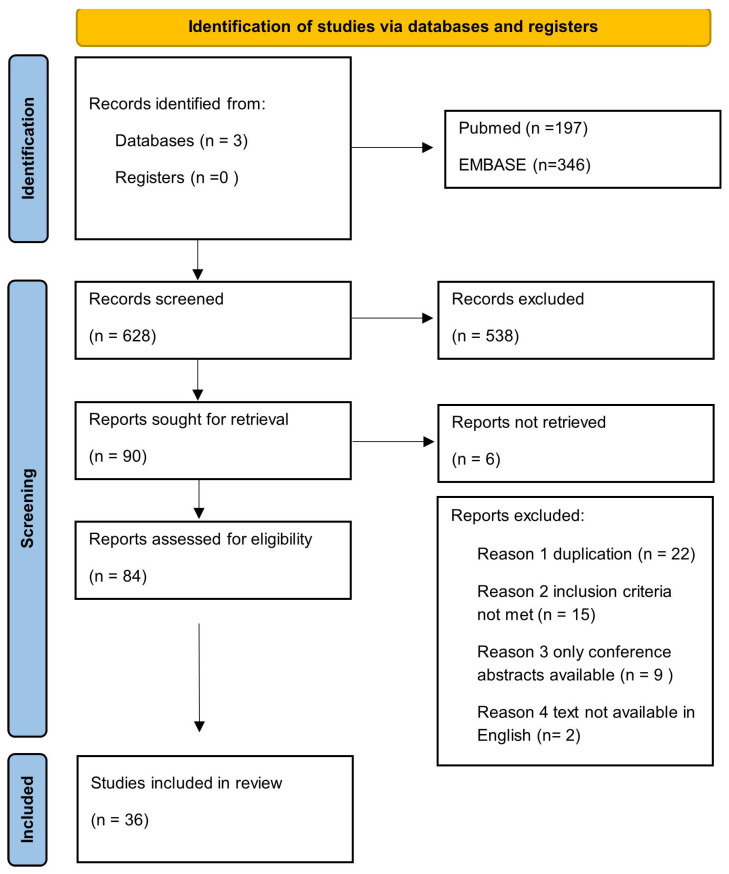
PRISMA flow diagram of study selection.

In the final synthesis, we included 36 studies which fulfilled all eligibility criteria published up to 30 April 2025. The whole process of study identification, screening and inclusion is summarised in the PRISMA flow diagram (Figure 1) [29,30,31,32,33,34,35,36,37,38,39,40,41,42,43,44,45,46,47,48,49,50,51,52,53,54,55,56,57,58,59,60,61,62,63,64]. A summary of the included studies and their basic characteristics is provided in Supplementary Materials S3.

### 3.2. General Information

This systematic review incorporated 36 preclinical studies published between 2009 and 2025, all of which were conducted using rodent models (thirty-one studies on mice, five studies on rats). In murine models, the C57BL/6 lineage (including J, N, and unspecified substrains) was the most frequently employed, followed by Kunming and BALB/c strains. Among rat models, Sprague–Dawley, Wistar, and Charles Foster albino rats were used. Sample sizes varied substantially, ranging from 12 to 96 animals per study (mean ≈ 44; median = 40). Six studies did not report the total sample size.

#### 3.2.1. Alcohol Induction Protocols

Alcohol administration protocols differed widely in both duration and delivery method. Chronic exposure models (typically 10–12 weeks, occasionally up to 3 months) were the most prevalent, although shorter acute or subacute regimens of 7–15 days were also employed. The Lieber–DeCarli liquid diet was the most frequently used approach, often supplemented by a single high-dose gavage on the last day to model “chronic-plus-binge” exposure.

#### 3.2.2. Probiotic Intervention

Probiotic treatment constituted the primary intervention and exhibited substantial taxonomic and methodological diversity across the included studies. Most investigations focused on bacteria from the genus *Lactobacillus*, with *Lactobacillus rhamnosus* being the most frequently studied species (n = 12 studies, including seven using the *L. rhamnosus GG* strain), followed by *Lactobacillus plantarum* (n = 8). Other *Lactobacillus* species were examined in an additional seven studies. *Bifidobacterium* species were investigated in four studies, while *Akkermansia muciniphila* was evaluated in two. Single studies assessed other genera, including *Acetobacter*, *Enterococcus*, *Lactococcus*, *Komagataeibacter*, *Pediococcus*, *Bacteroides*, and a genetically modified *Escherichia coli* strain.

Probiotic dosing regimens varied widely between studies and were reported using different units, including CFU/mL, CFU/day, and CFU/kg. Administered doses ranged from 2.5 × 10^7^ to 1 × 10^10^ CFU per day, with 1 × 10^9^ CFU/day being the most commonly used dose, suggesting an emerging standard for preclinical ALD models. Probiotics were predominantly administered via the oral route, either by gavage or mixed with drinking water or feed, reflecting clinically relevant exposure pathways. Treatment duration differed substantially across studies, typically ranging from several weeks to the entire period of alcohol exposure. This heterogeneity in intervention timing may contribute to variability in observed outcomes and should be considered when interpreting the results and translating them to the design of future human studies.

### 3.3. Impact of Probiotics on Alcohol-Induced Liver Injury

#### 3.3.1. Liver Injury Markers

Alcohol exposure consistently induced significant elevations in serum ALT and AST levels across all experimental models, confirming hepatocellular injury. Probiotic supplementation mitigated these biochemical alterations in the vast majority of studies, with most strains reducing ALT and/or AST levels toward reference values. Across 31 studies evaluating serum transaminases and encompassing 38 probiotic strains, reductions in ALT and AST activity were frequently substantial, reaching approximately 25–70% compared with alcohol-fed control groups in several models, including those using *Bifidobacterium animalis*, *Lactobacillus plantarum*, and *Lactobacillus rhamnosus GG*.

Despite this overall consistency, strain-specific differences were observed. Four probiotic strains did not demonstrate a clear hepatoprotective effect on transaminase levels: *Lactobacillus paracasei*, *Bifidobacterium bifidum*, and *Lactobacillus plantarum* and *Lactobacillus rhamnosus GG*, with the latter two showing reductions in AST but not ALT [38,42,43]. Moreover, supplementation with *Komagataeibacter hansenii* and *Enterococcus faecium* was associated with increased AST levels, further highlighting strain-dependent variability in biochemical outcomes [37,62].

#### 3.3.2. Histopathological Findings

Alcohol exposure consistently induced characteristic histopathological features of liver injury, including hepatic steatosis, hepatocyte ballooning, lobular disorganization, and inflammatory cell infiltration. Across 31 studies assessing liver histology and encompassing 36 probiotic strains, probiotic supplementation generally mitigated these pathological changes, resulting in reduced lipid accumulation, attenuation of inflammatory features, and partial restoration of normal hepatic architecture.

Despite this overall pattern of histological improvement, strain-specific differences were observed. Three probiotic strains—*Lactobacillus fermentum*, *Lactobacillus plantarum*, and *Escherichia coli* Ecn-3—did not demonstrate beneficial effects on histopathological outcomes, indicating that structural liver protection was not uniform across all probiotic interventions [43,53,61].

#### 3.3.3. Effects on Oxidative Stress and Antioxidant Enzymes

Ten studies evaluated the effects of alcohol feeding and probiotic intervention on hepatic oxidative stress and antioxidant enzyme activity. Alcohol-induced oxidative stress was evident through decreased hepatic levels or activity of superoxide dismutase (SOD), catalase (CAT), and glutathione (GSH), alongside elevated malondialdehyde (MDA) levels. Probiotic administration consistently restored antioxidant capacity, elevating SOD, CAT and GSH while reducing MDA, in some cases by up to 50%.

#### 3.3.4. Modulation of Inflammatory Pathways

Six studies evaluated hepatic mRNA expression of inflammatory cytokines, and thirteen measured circulating cytokine levels. Alcohol exposure was associated with a strong pro-inflammatory response, as reflected in the upregulation of TNF-α, IL-1β, and IL-6, as well as the activation of the NF-κB signalling pathway. Probiotic treatment suppressed these responses across multiple models. For instance, *L. plantarum* reduced TNF-α expression by more than 60% relative to alcohol-only controls. Such modulation of cytokine signalling likely contributes to the overall attenuation of hepatic inflammation.

The data summarising these findings are presented in Table 1, in the form of a heatmap illustrating relative changes in biochemical and histological outcomes across all included studies.

### 3.4. Probiotic Efficacy in Mitigating Alcohol-Induced Hepatic Steatosis

#### 3.4.1. Histopathological Assessment of Steatosis

Increased hepatic steatosis was found in vast majority of studies in alcohol-fed groups. Probiotic supplementation had a protective effect, reducing steatosis and improving hepatic architecture in nearly all studies that assessed histology. Quantitative analyses also supported these observations: *L. fermentum* reduced steatosis from grades II–III to grade I, and *L. brevis* and *B. thetaiotaomicron* both significantly lowered the steatosis in the liver [47,54,61].

#### 3.4.2. Modulation of Systemic and Hepatic Lipid Content

Alcohol exposure consistently disrupted lipid metabolism across experimental models, leading to increased serum triglycerides (TG), total cholesterol (TC), and low-density lipoprotein cholesterol (LDL-C), accompanied by reduced high-density lipoprotein cholesterol (HDL-C), as well as parallel increases in hepatic TG and TC content. Across the included studies, probiotic supplementation generally ameliorated these systemic and hepatic lipid disturbances, resulting in a more favourable lipid profile. In 18 studies assessing serum lipid parameters and encompassing 22 probiotic strains, most interventions were associated with reductions in circulating TG, TC, and LDL-C and, in several cases, increases in HDL-C. Consistent improvements in intrahepatic lipid content were observed in 12 studies evaluating hepatic lipid outcomes across 20 probiotic strains, indicating enhanced hepatic lipid metabolism.

Despite this overall pattern of benefit, strain-specific differences were evident. No improvement in serum lipid parameters was reported for *Lactobacillus plantarum* or *Escherichia coli* Ecn-2 and Ecn-3 [43,53]. Similarly, four strains did not demonstrate lipid-lowering effects in hepatic tissue (*E. coli* Ecn-2 and Ecn-3, *L. plantarum* from study by Tian et al., and *Lactobacillus paracasei* from Lv et al.) [38,43,53]. Notably, supplementation with *Lactobacillus sakei* and *Lactobacillus delbrueckii* was associated with increased hepatic triglyceride concentrations, highlighting that probiotic effects on lipid metabolism are strain dependent and, in some cases, potentially adverse [38]. A comprehensive overview of strain-specific effects on systemic and hepatic lipid markers is provided in Table 2 and Table 3.

### 3.5. Effects on the Gut Barrier and Endotoxemia

#### Gut Barrier

Alcohol exposure consistently impaired intestinal barrier integrity across experimental models, as evidenced by downregulation of tight junction proteins, including claudin-1, occludin, and zonula occludens-1 (ZO-1), leading to increased intestinal permeability. Across 16 studies assessing tight junction protein expression and encompassing 17 probiotic strains, probiotic supplementation generally restored barrier integrity, as reflected by increased expression of these proteins. In addition, selected studies reported upregulation of Muc2, the principal mucin component of the intestinal mucus layer, and functional improvements confirmed by reduced FITC–dextran permeability and recovery of villus architecture and epithelial integrity [32,34].

Despite this overall pattern, strain-specific differences were observed. Two probiotic strains did not demonstrate consistent improvements in tight junction protein expression: *Lactobacillus paracasei*, which did not increase claudin-1 or occludin levels, and *Lactobacillus reuteri*, which increased zonulin expression but not occludin [38,42].

Consistent with improvements in gut barrier function, endotoxin levels were reduced following probiotic supplementation. Across 21 studies evaluating circulating lipopolysaccharide (LPS) or related endotoxin markers and encompassing 24 probiotic strains, all reported reductions in systemic endotoxemia, indicating a highly consistent effect of probiotics on alcohol-induced gut-derived endotoxin translocation.

A comprehensive overview of these findings is provided in Table 4.

### 3.6. The Impact of Alcohol on the Gut Microbiota

Alcohol exposure consistently disrupted the gut microbial homeostasis, producing a distinct dysbiotic signature characterised by reduced microbial diversity and compositional imbalance. Measures of α-diversity (ACE, Chao1 and Shannon indices), if evaluated, were decreased in most studies.

At the phylum level, alcohol feeding typically led to an expansion of *Proteobacteria*, a phylum rich in LPS-producing, pro-inflammatory species, accompanied by a decline in *Bacteroidetes*. Changes in *Firmicutes* were more variable, with both increases and decreases reported depending on model conditions.

At the genus level, alcohol exposure promoted the proliferation of opportunistic taxa, such as *Escherichia–Shigella*, *Helicobacter*, *Alistipes*, and *Mucispirillum*, while depleting beneficial genera, including *Lactobacillus* and *Akkermansia*. Collectively, these shifts indicate a consistent trend toward a less diverse, more pro-inflammatory microbial community.

### 3.7. The Effects of Probiotic Intervention on the Gut Microbiota

Probiotic intervention counteracted the dysbiotic effects of alcohol, restoring both microbial diversity and taxonomic balance. Across multiple studies, probiotics increased α-diversity indices. Strains such as *Acetobacter pasteurianus* BP2201, *Bacillus coagulans* BC01, and *Bifidobacterium breve* ATCC 15700 significantly increased the Chao1 and Shannon indices.

At the taxonomic level, probiotics consistently reduced the abundance of *Proteobacteria* while restoring the abundance of Firmicutes and Bacteroidetes, thereby reversing the alcohol-induced shift in phylum-level abundance. For example, *Akkermansia muciniphila* and *Lactobacillus rhamnosus* GG both decreased *Proteobacteria* while increasing beneficial phyla. Probiotics suppressed potentially pathogenic taxa (for example, *Escherichia–Shigella* and *Helicobacter*) and promoted beneficial genera, such as *Lactobacillus*, *Akkermansia*, and *Turicibacter*.

The data summarising the changes to the gut microbiota at the phylum levels, as well as to the diversity indices, are presented in Table 5, which displays the directional effects of alcohol and probiotic interventions.

### 3.8. Risk of Bias Analysis

The methodological quality of the included studies was assessed using SYRCLE’s risk of bias tool by two independent reviewers (K.S. and R.K.) [13]. The detailed results of the risk of bias assessment are provided in Supplementary Materials S4.

Overall, the risk of bias varied across studies, with several domains frequently rated as unclear due to insufficient reporting. While most studies adequately described baseline characteristics and outcome assessment, details regarding random sequence generation, allocation concealment, and blinding of investigators were often lacking. Performance and detection bias could therefore not be reliably excluded in a substantial proportion of studies.

Using the GRADE framework, the overall certainty of evidence for the critical outcomes was rated as very low. The evidence was downgraded by one level for serious risk of bias, reflecting the widespread methodological and reporting limitations identified by the SYRCLE assessment. A further two-level downgrade was applied for very serious indirectness, as all evidence originated from rodent models of alcohol-associated liver disease, limiting applicability to human clinical settings. Additional downgrading was applied for serious imprecision due to incomplete numerical outcome reporting—quantitative data for key outcomes such as serum ALT and AST activity with measures of variability were available in only a small subset of studies—precluding meta-analysis and precise estimation of effect size. Finally, the certainty of evidence was downgraded for suspected publication bias, given the predominance of positive findings and the inability to formally assess publication bias.

Formal evaluation of publication bias using funnel plots or statistical tests was not feasible because most studies lacked quantitative effect estimates and measures of variance. Nevertheless, the potential impact of publication bias was considered qualitatively and incorporated into the GRADE assessment.

## 4. Discussion

This systematic review synthesises sixteen years of preclinical evidence (36 studies) investigating the hepatoprotective effects of probiotics in animal models of alcohol-induced liver injury. In reviewed studies, probiotic intervention consistently ameliorated biochemical, histological, and molecular hallmarks of ALD. The results support a consistent mechanistic model: probiotics attenuate ethanol-induced hepatic injury by rebalancing the gut microbiota, restoring intestinal barrier integrity, reducing systemic endotoxemia, and suppressing oxidative and inflammatory cascades within the liver. Collectively, these findings strongly suggest that the gut–liver axis represents an important therapeutic target in ALD and point to probiotics as a promising avenue for future intervention.

Ethanol-induced dysbiosis is a well-recognised consequence of alcohol intake. It reduces microbial diversity and shifts the gut microbiota towards a pro-inflammatory profile, depleting beneficial *Firmicutes* and *Bacteroidetes* while enriching *Proteobacteria* [65]. Studies included in this review consistently reproduced these patterns. The shift in gut microbiota composition disrupts intestinal homeostasis, leading to impaired barrier function and subsequent endotoxemia [7]. Alcohol exposure markedly decreased the expression of tight-junction proteins, including Claudin-1, Occludin, and ZO-1. In contrast, probiotic supplementation tended to restore or even enhance their expression, in some cases even exceeding control levels. These changes closely corresponded with alterations in circulating lipopolysaccharide and other bacteria-derived endotoxins. Increased concentrations of bacterial endotoxins in the systemic and especially portal circulation are well-established drivers of hepatic injury in ALD [66]. In the study by Lv et al., supplementation with *Lactobacillus paracasei* failed to improve the expression of the tight-junction proteins Claudin-1 and Occludin, although it did reduce circulating LPS levels. Despite this partial effect, the *L. paracasei* group showed no improvement in serum ALT or AST concentrations. In contrast, treatment with *Lactobacillus helveticus* in the same study restored the expression of Claudin-1 and Occludin, and significantly lowered ALT and AST levels [38]. These findings suggest that preservation of tight-junction integrity is an important mechanism underlying the hepatoprotective effects of probiotics in ALD.

Inflammatory modulation emerged as another consistent finding. Alcohol exposure elevated hepatic and systemic levels of pro-inflammatory cytokines such as TNF-α, IL-1β, and IL-6, while reducing the anti-inflammatory cytokine IL-10. This cytokine profile mirrors the hepatic inflammation characteristic of ALD [67]. Probiotic supplementation effectively reversed these changes, further demonstrating its ability to mitigate liver inflammation.

Ethanol exerted a consistently detrimental effect on liver tissue, as evidenced by elevated serum ALT and AST, extensive hepatocyte degeneration, steatosis, and inflammatory infiltration. Probiotic treatment largely prevented or attenuated the development of these lesions. In parallel, alcohol exposure induced oxidative stress within the liver, evidenced by decreased activity of antioxidant enzymes (SOD, CAT, and GSH) and increased lipid peroxidation markers. Probiotic supplementation restored antioxidant capacity, normalising enzyme activity and reducing oxidative damage. This restoration of redox balance is particularly relevant, as oxidative stress represents a well-established pathogenic mechanism in the development of ALD [68].

At the metabolic level, alcohol enhances fatty acid uptake, suppresses β-oxidation, and stimulates hepatic lipogenesis, ultimately leading to the development of steatosis [69]. Probiotic treatment counteracted these effects across several models included in this review, resulting in normalisation of serum and hepatic triglyceride levels, thereby preventing or alleviating hepatic steatosis.

This review has several strengths, including a pre-registered protocol, adherence to PRISMA guidelines, and the use of SYRCLE’s risk of bias tool and GRADE framework to appraise methodological quality systematically. However, several limitations—inherent primarily to the primary literature—should be acknowledged. First, the included studies showed substantial heterogeneity in animal species and strains, alcohol-induction protocols, probiotic strains and dosing regimens, as well as outcome measures. Second, outcome reporting was frequently incomplete. Numerical data (for example, means, standard deviations, or exact effect sizes) were often absent, with results described only qualitatively (for example, increased or significantly reduced) or presented solely in graphical form without extractable values. In some cases, key outcomes were mentioned in the text without corresponding data, and selective reporting of positive findings cannot be excluded. These factors reduce the transparency and reproducibility of the evidence base, potentially biasing our synthesis towards outcomes that are more clearly reported. Incomplete data and variability prevented us from performing a quantitative meta-analysis, requiring a predominantly narrative synthesis, which limits the precision with which effect sizes can be estimated. Third, methodological quality was variable: several studies provided limited detail on randomisation, allocation concealment and blinding, and many were underpowered, with small sample sizes. Fourth, six potentially eligible reports could not be retrieved despite attempts to contact the corresponding authors, which may have influenced the completeness of the evidence base. Finally, all data were derived from rodent models, which, although highly informative mechanistically, cannot fully recapitulate the complexity of human ALD, including co-morbidities, patterns of alcohol use and dietary influences. These limitations should be considered when applying our findings to clinical practice.

## 5. Conclusions

This systematic review synthesizes the available preclinical evidence indicating that probiotic supplementation may ameliorate multiple facets of alcohol-induced liver injury. Across a wide range of experimental models and probiotic strains, probiotics were generally associated with improvements in biochemical markers of liver injury, attenuation of steatosis, restoration of antioxidant defences, and reduction in hepatic inflammation. At the level of the gut–liver axis, probiotic interventions were linked to reversal of alcohol-induced dysbiosis, reinforcement of intestinal barrier integrity, reductions in endotoxin burden, and modulation of key pathogenic pathways involved in liver injury.

However, when formally evaluated using the GRADE framework, the overall certainty of evidence for the critical outcomes was rated as very low, primarily due to methodological limitations, indirectness inherent to animal models, imprecision of outcome reporting, and suspected publication bias. Consequently, these findings should be interpreted with caution, and direct extrapolation to human disease is not warranted. While the preclinical data provide a coherent mechanistic rationale and support further investigation, they should primarily be viewed as hypothesis-generating and as a foundation for the design of well-controlled, adequately powered clinical trials evaluating carefully characterised probiotic strains as adjunctive therapies in alcohol-associated liver disease.

## Figures and Tables

**Table 1:** The effects of probiotic treatment on ALT and AST activity and hepatocellular damage in alcohol-induced liver injury [29,30,31,32,33,34,35,36,37,38,39,40,41,42,43,44,45,46,47,48,49,50,51,52,53,54,55,56,57,58,59,60,61,62,63,64]. ALT—Alanine Aminotransferase, AST—Aspartate Aminotransferase, n.d.—no data.

Parameter	*Acetobacter pasteurianus* BP2201, Wen et al., 2023 [29]		*Akkermansia municiphila*, Fang et al., 2023 [30]		*Lactobacillus fermentum,* Barone et al., 2016 [61]		*Lactobacillus plantarum* ZS62, Gan et al., 2021 [31]		*Bacillus coagulans* BC01, Liu et al., 2024 [32]		*Bifidobacterium breve* ATCC15700, Tian et al., 2020 [33]		*Bacillus subtilis* (CMCC 1.3358), Zhao et al., 2021 [34]		*Lactobacillus plantarum* (MTCC 1325), Arora et al., 2014 [35]		*Lactobacillus rhamnosus* GG, Shi et al., 2015 [60]		*Lactococcus chungangensis* CAU 1447, Nam et al., 2019 [36]		*Komagataeibacter hansenii* CGMCC 3917, Lin et al., 2020 [37]		*Lacticaseibacillus rhamnosus* CCFM1060, Niu et al., 2024 [63]		*Lacticaseibacillus rhamnosus* LRa05., Gu et al., 2024 [39]		*Lactobacillus* GG, Forsyth et al., 2009 [64]		*Lactobacillus helveticus* (and Others), Lv et al., 2024 [38]							*Lactobacillus plantarum* J26, Li et al., 2024 [41]		*Lactobacillus plantarum* J26, Li et al., 2022 [40]		*Bifidobacterium bifidum* YRT3115, Zhang et al., 2019 [42]		
	Alcohol	Probiotic	Alcohol	Probiotic	Alcohol	Probiotic	Alcohol	Probiotic	Alcohol	Probiotic	Alcohol	Probiotic	Alcohol	Probiotic	Alcohol	Probiotic	Alcohol	Probiotic	Alcohol	Probiotic	Alcohol	Probiotic	Alcohol	Probiotic	Alcohol	Probiotic	Alcohol	Probiotic	Alcohol		*Lactobacillus helveticus*	*Lactobacillus casei*	*Lactobacillus paracasei*	*Lactobacillus sakei*	*Lactobacillus delbrueckii*	Alcohol	Probiotic	Alcohol	Probiotic	Alcohol	*B. bifidum*	*B. animalis* subsp. Lactis
ALT	↑	↓	↑	↓	↑	↓	↑	↓	↑	↓	↑	↓	↑	↓	↑	↓	n.d.	n.d.	↑	↓	↑	↓	↑	↓	↑	↓	n.d.	n.d.	↑		↓	↓	↔	↓	↓	n.d.	n.d.	↑	↓	↑	↔	↓
AST	↑	↓	↑	↓	↑	↓	↑	↓	↑	↓	↑	↓	↑	↓	↑	↓	n.d.	n.d.	↑	↓	↑	↓	↑	↓	↑	↓	n.d.	n.d.	↑		↓	↓	↔	↓	↓	n.d.	n.d.	↑	↓	↑	↔	↓
Hepatocellular damage and inflammation	↑	↓	↑	↓	↔	↔	↑	↓	n.d.	n.d.	↑	↓	↑	↓	↑	↓	n.d.	n.d.	↑	↓	↑	↓	↑	↓	↑	↓	↑	↓	↑		↓	↓	↓	↓	↓	↑	↓	↑	↓	n.d.	n.d.	n.d.
	***Lactobacillus rhamnosus* GG,** **Wang et al., 2013** [59]		***Lactobacillus rhamnosus* GG,** **Wang et al. 2011** [44]		***Lactobacillus rhamnosus* GG (ATCC 53103),** **Gu et al., 2020** [45]				***Lactobacillus rhamnosus* NKU FL1-8,** **Liu et al. 2024** [46]		***Levilactobacillus brevis* MG5311,** **Jung et al., 2022** [47]		***Lactobacillus reuteri,*** **Zheng et al. 2020** [48]		***Lactobacillus rhamnosus* GG,** **Bull-Otterson et al., 2013** [49]		***Pediococcus pentosaceus* CGMCC 7049,** **Jiang et al. 2020** [50]		***Lactobacillus plantarum* CMU995 (DSM 23780),** **Fang et al., 2019** [51]		***Lactobacillus rhamnosus* CCFM1107** **Tian et al., 2015** [43]				***Escherichia coli* Nissle 1917 (EcN),** **Singh et al., 2014** [53]				***Bacteroides thetaiotaomicron* DSM 2079,** **Sangineto et al., 2022** [54]		***Bifidobacterium animalis* F1-7,** **Liang et al. 2024** [55]		***Lactobacillus rhamnosus* GG,** **Ge et al. 2022** [56]		***Lactobacillus rhamnosus* B10,** **Li et al., 2022** [57]		***Lactobacillus plantarum* FB091,** **Lee et al. 2024** [58]		***Akkermansia muciniphila*, ATCC,** **Wei et al. 2024** [52]		***Lactobacillus rhamnosus* GG,** **Wang et al., 2013** [59]	
	Alcohol	Probiotic	Alcohol	Probiotic	Alcohol	Low dose	Medium dose	High dose	Alcohol	Probiotic	Alcohol	Probiotic	Alcohol	Probiotic	Alcohol	Probiotic	Alcohol	Probiotic	Alcohol	Probiotic	Alcohol	Lactobacillus rhamnosus CCFM1107	Lactobacillus plantarum CCFM1112	Lactobacillus rhamnosus GG	Alcohol	EcN-2	EcN-3	EcN-4	Alcohol	Probiotic	Alcohol	Probiotic	Alcohol	Probiotic	Alcohol	Probiotic	Alcohol	Probiotic	Alcohol	Probiotic	Alcohol	Probiotic
ALT	n.d.	n.d.	↑	↓	↑	↓	↓	↓	↑	↓	↑	↓	↑	↓	↑	↓	↑	↓	↑	↓	↑	↓	↔	↔	n.d.	n.d.	n.d.	n.d.	↑	↔	↑	↓	↑	↓	↑	↓	↑	↓	↑	↓	↑	↓
AST	n.d.	n.d.	n.d.	n.d.	↑	↓	↓	↓	↑	↓	↑	↓	↑	↓	↑	↓	↑	↓	↑	↓	↑	↓	↔	↓	n.d.	n.d.	n.d.	n.d.	n.d.	n.d.	↑	↓	↑	↓	↑	↓	↑	↓	↑	↓	↑	↑
Hepatocellular damage and inflammation	↑	↓	↑	↓	↑	↓	↓	↓	↑	↓	↑	↓	↑	↓	↑	↓	↑	↓	↑	↓	↑	↓	↔	↓	↑	n.d.	↔	↓	n.d.	n.d.	↑	↓	↑	↓	n.d.	n.d.	↑	↓	↑	↓	↑	↓

**Table 2:** The effects of alcohol and probiotic treatment on serum lipid profiles [29,31,33,34,36,37,39,40,41,43,46,47,48,53,54,55,57,63]. TC—total cholesterol, TG—triglycerides, n.d.—no data.

Parameter	*Acetobacter pasteurianus* BP2201, Wen et al., 2023 [29]		*Lactobacillus plantarum* ZS62, Gan et al., 2021 [31]		*Bifidobacterium breve* ATCC15700, Tian et al., 2020 [33]		*Bacillus subtilis* (CMCC 1.3358), Zhao et al., 2021 [34]		*Lactococcus chungangensis* CAU 1447, Nam et al., 2019 [36]		*Komagataeibacter hansenii* CGMCC 3917, Lin et al., 2020 [37]		*Lacticaseibacillus rhamnosus* CCFM1060, Niu et al., 2024 [63]		*Lacticaseibacillus rhamnosus* LRa05., Gu et al., 2024 [39]		*Lactobacillus plantarum* J26, Li et al., 2024 [41]		*Lactobacillus plantarum* J26, Li et al., 2022 [40]		*Lactobacillus rhamnosus* CCFM1107 Tian et al., 2015 [43]				*Lactobacillus rhamnosus* NKU FL1-8, Liu et al. 2024 [46]		*Levilactobacillus brevis* MG5311, Jung et al., 2022 [47]		*Lactobacillus reuteri,* Zheng et al. 2020 [48]		*Escherichia coli* Nissle 1917 (EcN), Singh et al., 2014 [53]				*Bacteroides thetaiotaomicron* DSM 2079, Sangineto et al., 2022 [54]		*Bifidobacterium animalis* F1-7, Liang et al. 2024 [55]		*Lactobacillus rhamnosus* B10, Li et al., 2022 [57]	
	Alcohol	Probiotic	Alcohol	Probiotic	Alcohol	Probiotic	Alcohol	Probiotic	Alcohol	Probiotic	Alcohol	Probiotic	Alcohol	Probiotic	Alcohol	Probiotic	Alcohol	Probiotic	Alcohol	Probiotic	Alcohol	*L. rhamnosus CCFM1107*	*L. plantarum CCFM1112*	*L. rhamnosus* GG	Alcohol	Probiotic	Alcohol	Probiotic	Alcohol	Probiotic	Alcohol	EcN-2	EcN-3	EcN-4	Alcohol	Probiotic	Alcohol	Probiotic	Alcohol	Probiotic
Serum TC	↑	↓	↑	↓	n.d.	n.d.	↑	↓	n.d.	n.d.	n.d.	n.d.	↑	↓	n.d.	n.d.	↑	↓	↑	↓	↑	↓	↔	↓	↑	↓	↓	↑	↑	↓	↑	↔	↔	↓	n.d.	n.d.	↑	↓	↑	↓
Serum TG	↑	↓	↑	↓	↑	↓	n.d.	n.d.	↑	↓	↑	↓	↑	↓	↑	↓	↑	↓	↑	↓	↑	↓	↔	↓	↑	↓	↓	↑	↑	↓	↑	↔	↔	↓	↑	↓	↑	↓	↑	↓

**Table 3:** The effects of alcohol and probiotic treatment on hepatic lipid profiles [30,32,34,38,39,43,44,51,54,57,63]. TC—total cholesterol, TG—triglycerides, n.d.—no data.

Parameter	*Akkermansia municiphila*, Fang et al., 2023 [30]		*Bacillus coagulans* BC01, Liu et al., 2024 [32]		*Bacillus subtilis* (CMCC 1.3358), Zhao et al., 2021 [34]		*Lacticaseibacillus rhamnosus* LRa05., Gu et al., 2024 [39]		*Lactobacillus rhamnosus* GG (ATCC 53103), Gu et al., 2020 [45]				*Escherichia coli* Nissle 1917 (EcN), Singh et al., 2014 [53]				*Lactobacillus plantarum* CMU995 (DSM 23780), Fang et al., 2019 [51]		*Lactobacillus rhamnosus* CCFM1107 Tian et al., 2015 [43]				*Lactobacillus rhamnosus* GG, Wang et al. 2011 [44]		*Lactobacillus helveticus* (and Others), Lv et al., 2024 [38]						*Lactobacillus rhamnosus* B10, Li et al., 2022 [57]		*Bacteroides thetaiotaomicron* DSM 2079, Sangineto et al., 2022 [54]	
	Alcohol	Probiotic	Alcohol	Probiotic	Alcohol	Probiotic	Alcohol	Probiotic	Alcohol	Low dose	Medium dose	High dose	Alcohol	EcN-2	EcN-3	EcN-4	Alcohol	Probiotic	Alcohol	*L. rhamnosus* CCFM1107	*L. plantarum* CCFM1112	*L. rhamnosus* GG	Alcohol	Probiotic	Alcohol	*L. helveticus*	*L. casei*	*L. paracasei*	*L. sakei*	*L. delbrueckii*	Alcohol	Probiotic	Alcohol	Probiotic
Hepatic TC	n.d.	n.d.	n.d.	n.d.	n.d.	n.d.	n.d.	n.d.	n.d.	n.d.	n.d.	n.d.	↑	↔	↔	↓	↑	↓	↑	↓	↔	↓	↑	↓	n.d.	n.d.	n.d.	n.d.	n.d.	n.d.	n.d.	n.d.	n.d.	n.d.
Hepatic TG	↓	↑	↑	↓	↑	↓	↑	↓	↑	↓	↓	↓	↑	↔	↔	↓	↑	↓	↑	↓	↔	↓	↑	↓	↑	↓	↓	↔	↑	↑	↑	↓	↑	↓

**Table 4:** The effect of alcohol and probiotic intervention on tight-junction protein expression and endotoxin levels [30,32,33,34,35,37,38,39,40,41,43,44,45,46,48,49,50,51,52,54,55,57,63,64], n.d.—no data.

Parameter		Claudin-1	Occludin	ZO-1	Endotoxins
*Akkermansia municiphila*, Fang et al., 2023 [30]	Alcohol	n.d.	↓	↓	n.d.
	Probiotic	n.d.	↑	↑	n.d.
*Bacillus coagulans* BC01, Liu et al., 2024 [32]	Alcohol	↓	↓	↓	↑
	Probiotic	↑	↑	↑	↓
*Bifidobacterium breve* ATCC15700, Tian et al., 2020 [33]	Alcohol	↓	↓	↓	↑
	Probiotic	↑	↑	↑	↓
*Bacillus subtilis* (CMCC 1.3358), Zhao et al., 2021 [34]	Alcohol	n.d.	↓	↓	↑
	Probiotic	n.d.	↑	↑	↓
*Lactobacillus plantarum* (MTCC 1325), Arora et al., 2014 [35]	Alcohol	n.d.	n.d.	n.d.	↑
	Probiotic	n.d.	n.d.	n.d.	↓
*Komagataeibacter hansenii* CGMCC 3917, Lin et al., 2020 [37]	Alcohol	n.d.	n.d.	n.d.	↑
	Probiotic	n.d.	n.d.	n.d.	↓
*Lacticaseibacillus rhamnosus* CCFM1060, Niu et al., 2024 [63]	Alcohol	↓	↓	↓	n.d.
	Probiotic	↑	↑	↑	n.d.
*Lacticaseibacillus rhamnosus* LRa05., Gu et al., 2024 [39]	Alcohol	n.d.	n.d.	n.d.	↑
	Probiotic	n.d.	n.d.	n.d.	↓
*Lactobacillus* GG, Forsyth et al., 2009 [64]	Alcohol	↓	↓	n.d.	n.d.
	Probiotic	↑	↑	n.d.	n.d.
*Lactobacillus helveticus* (and Others), Lv et al., 2024 [38]	Alcohol	↓	↓	n.d.	↑
	*Lactobacillus helveticus* (LH, ATCC 15009)	↑	↑	n.d.	↓
	*Lactobacillus casei* (LC, ATCC 393)	n.d.	n.d.	n.d.	n.d.
	*Lactobacillus paracasei* (LP, ATCC 25302)	↔	↔	n.d.	↓
	*Lactobacillus sakei* (LS, ATCC 15521)	n.d.	n.d.	n.d.	n.d.
	*Lactobacillus delbrueckii* (LD, ATCC 9649)	n.d.	n.d.	n.d.	n.d.
*Lactobacillus plantarum* J26, Li et al., 2024 [41]	Alcohol	n.d.	n.d.	n.d.	↑
	Probiotic	n.d.	n.d.	n.d.	↓
*Lactobacillus plantarum* J26, Li et al., 2022 [40]	Alcohol	↓	↓	↓	↑
	Probiotic	↑	↑	↑	↓
*Lactobacillus rhamnosus* CCFM1107 Tian et al., 2015 [43]	Alcohol	n.d.	n.d.	n.d.	↑
	*Lactobacillus rhamnosus* CCFM1107	n.d.	n.d.	n.d.	↓
	*Lactobacillus plantarum* CCFM1112	n.d.	n.d.	n.d.	↓
	*Lactobacillus rhamnosus* GG	n.d.	n.d.	n.d.	↓
*Lactobacillus rhamnosus* GG, Wang et al. 2011 [44]	Alcohol	↓	↓	↓	↑
	Probiotic	↑	↑	↑	↓
*Lactobacillus rhamnosus* GG (ATCC 53103), Gu et al., 2020 [45]	Alcohol	n.d.	n.d.	n.d.	↑
	Low dose	n.d.	n.d.	n.d.	↓
	Medium dose	n.d.	n.d.	n.d.	↓
	High dose	n.d.	n.d.	n.d.	↓
*Lactobacillus rhamnosus* NKU FL1-8, Liu et al. 2024 [46]	Alcohol	n.d.	↓	↓	↑
	Probiotic	n.d.	↑	↑	↓
*Lactobacillus reuteri*, Zheng et al. 2020 [48]	Alcohol	n.d.	↔	↓	↑
	Probiotic	n.d.	↔	↑	↓
*Lactobacillus rhamnosus* GG, Bull-Otterson et al., 2013 [49]	Alcohol	↓	↓	↓	↑
	Probiotic	↑	↑	↑	↓
*Pediococcus pentosaceus* CGMCC 7049, Jiang et al. 2020 [50]	Alcohol	n.d.	n.d.	↓	↑
	Probiotic	n.d.	n.d.	↑	↓
*Lactobacillus plantarum* CMU995 (DSM 23780), Fang et al., 2019 [51]	Alcohol	n.d.	n.d.	↓	↑
	Probiotic	n.d.	n.d.	↑	↓
*Bacteroides thetaiotaomicron* DSM 2079, Sangineto et al., 2022 [54]	Alcohol	↓	↓	↓	↑
	Probiotic	↑	↑	↑	↓
*Bifidobacterium animalis* F1-7, Liang et al. 2024 [55]	Alcohol	n.d.	n.d.	n.d.	↑
	Probiotic	n.d.	n.d.	n.d.	↓
*Lactobacillus rhamnosus* B10, Li et al., 2022 [57]	Alcohol	n.d.	n.d.	n.d.	↑
	Probiotic	n.d.	n.d.	n.d.	↓
*Akkermansia muciniphila*, ATCC, Wei et al. 2024 [52]	Alcohol	↓	↓	↓	↑
	Probiotic	↑	↑	↑	↓

**Table 5:** The effect of alcohol and probiotic interventions on the gut microbiota on the phylum level [29,30,32,33,34,37,38,40,45,46,49,50,52,57,58], n.d.—no data, *—trend not statistically significant.

Study		*Firmicutes*	*Bacteroidetes*	*Proteobacteria*
*Acetobacter pasteurianus* BP2201, Wen et al., 2023 [29]	Alcohol	↑	↓	↑
	*A. pasteurianus*	↔	↑	↓
*Akkermansia municiphila*, Fang et al., 2023 [30]	Alcohol	↓	↓	↑
	*A. muciniphila*	↑	↑	↓
*Bacillus coagulans* BC01, Liu et al., 2024 [32]	Alcohol	↓	↓	↑
	*B. coagulans*	↑	↑	↓
*Bifidobacterium breve* ATCC15700, Tian et al., 2020 [33]	Alcohol	↑	↓	↔
	*B. breve*	↓	↑	↓
*Bacillus subtilis* (CMCC 1.3358), Zhao et al., 2021 [34]	Alcohol	↓	↔	↑
	*B. subtlis*	↑	↔	↓
*Komagataeibacter hansenii* CGMCC 3917, Lin et al., 2020 [37]	Alcohol	↑	↓	↑
	*K. hansenii*	↓	↑	↓
*Lacticaseibacillus rhamnosus* LRa05., Gu et al., 2024 [39]	Alcohol	↓	↓	↑
	*L. rhamnosus* LRa05	↑	↑	↓ *
*Lactobacillus helveticus* (and others), Lv et al., 2024 [38]	Alcohol	↑	↑	↓
	*L. helveticus*	↔	↔	↔
	*L. casei*	n.d.	n.d.	n.d.
	*L. paracasei*	↓	↔	↓
	*L. sakei*	n.d.	n.d.	n.d.
	*L. delbrueckii*	n.d.	n.d.	n.d.
*Lactobacillus plantarum* J26, Li et al., 2022 [40]	Alcohol	↓	↑	↑
	*L. plantarum*	↑	↔	↓
*Lactobacillus rhamnosus* GG (ATCC 53103), Gu et al., 2020 [45]	Alcohol	↓	↔	↑
	Low dose	↑	↔	↓
	Medium dose	↔	↔	↑
	High dose	↑	↑	↓
*Lactobacillus rhamnosus* NKU FL1-8, Liu et al. 2024 [46]	Alcohol	↔	↔	↑
	*L. rhamnosus* NKU FL1-8	↑	↑	↓
*Lactobacillus rhamnosus* GG, Bull-Otterson et al., 2013 [49]	Alcohol	↓	↓	↑
	*L. rhamnosus* GG	↑	↔	↓
*Pediococcus pentosaceus* CGMCC 7049, Jiang et al. 2020 [50]	Alcohol	↑	↓	↔
	*P. penosaceus*	↓	↑	↓
*Bifidobacterium animalis* F1-7, Liang et al. 2024 [55]	Alcohol	↑	↑	↔
	*B. animalis*	↔	↔	↔
*Lactobacillus rhamnosus* B10, Li et al., 2022 [57]	Alcohol	↔	n.d.	↑
	*L. rhamnosus* B10	↑	n.d.	↓
*Lactobacillus plantarum* FB091, Lee et al. 2024 [58]	Alcohol	↔	↔	↔
	*L. plantarum* FB091	↔	↔	↓
*Akkermansia muciniphila*, ATCC, Wei et al. 2024 [52]	Alcohol	↑	↓	↑
	*A. muciniphila*	↓	↑	↓

## Data Availability

No new data were created or analyzed in this study. Data sharing is not applicable to this article.

## Supplementary Materials

The following supporting information can be downloaded at: https://www.mdpi.com/article/10.3390/nu18040608/s1, Supplementary Materials S1: Database queries; Supplementary Materials S2: Database; Supplementary Materials S3: List of studies; Supplementary Materials S4: Risk of Bias Analysis.

## Abbreviations

The following abbreviations are used in this manuscript:

ALD	Alcohol-associated liver disease
ALT	Alanine aminotransferase
AST	Aspartate aminotransferase
CAT	Catalase
CFU	Colony-forming units
GSH	Glutathione
HDL-C	High-density lipoprotein cholesterol
IL-1β	Interleukin 1 beta
IL-6	Interleukin 6
IL-10	Interleukin 10
LDL-C	Low-density lipoprotein cholesterol
LPS	Lipopolysaccharide
MDA	Malondialdehyde
mRNA	Messenger ribonucleic acid
Muc2	Mucin 2
NF-κB	Nuclear factor kappa B
SOD	Superoxide dismutase
TC	Total cholesterol
TG	Triglycerides
TNF-α	Tumor necrosis factor alpha
ZO-1	Zonula occludens-1
