# Supplementary material for: Efficacy of Probiotic Treatment in Alcoholic Liver Disease: A Systematic Review of Animal Studies
Source: Nutrients. 2026 Feb 12;18(4):608. doi: 10.3390/nu18040608 (PMC12943266; doi:10.3390/nu18040608)
Supplement: Supplementary file 1 [file nutrients-18-00608-s001.zip › Supplementary Material 1 Database queries.pdf]

Databases and Search Queries

The following search queries were used to identify relevant studies in PubMed, EMBASE, and AGRICOLA databases. The search strategy includes both controlled vocabulary terms (MeSH, Emtree) and free-text keywords to ensure comprehensive retrieval of relevant literature.

PubMed

("Liver Diseases, Alcoholic"[MeSH] OR "Ethanol/toxicity"[MeSH] OR "Ethanol/adverse effects"[MeSH] OR "Alcohol-Induced Liver Disease"[TIAB] OR "Alcoholic Liver Disease"[TIAB] OR "Alcohol Liver Injury"[TIAB])
AND
("Probiotics"[MeSH] OR "Gastrointestinal Microbiome"[MeSH] OR "Probiotic Therapy"[TIAB] OR "Gut Microbiota"[TIAB] OR "Gut Microbiome"[TIAB] OR "Intestinal Flora"[TIAB] OR "Microbiota Modulation"[TIAB] OR "Microbiome Diversity"[TIAB])
AND
("Animals"[MeSH] OR "Animal Experimentation"[MeSH] OR "Mice"[MeSH] OR "Rats"[MeSH] OR "Animal Models"[TIAB] OR "Experimental Models"[TIAB])
NOT
("Review"[Publication Type] OR "Systematic Review"[TIAB] OR "Meta-Analysis"[TIAB] OR "Narrative Review"[TIAB])
NOT
("Humans"[MeSH])

EMBASE

('alcoholic liver disease'/exp OR 'ethanol toxicity'/exp OR 'ethanol adverse effect'/exp OR 'alcohol-induced liver disease':ti,ab OR 'alcoholic liver disease':ti,ab OR 'alcohol liver injury':ti,ab)
AND
('probiotic agent'/exp OR 'gastrointestinal microbiota'/exp OR 'probiotic therapy':ti,ab OR 'gut microbiota':ti,ab OR 'gut microbiome':ti,ab OR 'intestinal flora':ti,ab OR 'microbiota modulation':ti,ab OR 'microbiome diversity':ti,ab)
AND
('animal experiment'/exp OR 'rodent'/exp OR 'mice'/exp OR 'rats'/exp OR 'animal model':ti,ab OR 'experimental model':ti,ab)
NOT
('review'/exp OR 'systematic review':ti,ab OR 'meta-analysis':ti,ab OR 'narrative review':ti,ab)
NOT
('human'/exp)

AGRICOLA

("alcoholic liver disease" OR "ethanol toxicity" OR "ethanol adverse effects" OR "alcohol induced liver disease" OR "alcoholic liver disease" OR "alcohol liver injury")
AND
("probiotics" OR "gastrointestinal microbiome" OR "probiotic therapy" OR "gut microbiota" OR "gut microbiome" OR "intestinal flora" OR "microbiota modulation" OR "microbiome diversity")
AND
("animals" OR "animal studies" OR "rodents" OR "mice" OR "rats" OR "animal models" OR "experimental models")
NOT
("review" OR "systematic review" OR "meta-analysis" OR "narrative review")
NOT
("human studies" OR "clinical trials" OR "human subjects")
