# Supplementary material for: Efficacy of Probiotic Treatment in Alcoholic Liver Disease: A Systematic Review of Animal Studies
Source: Nutrients. 2026 Feb 12;18(4):608. doi: 10.3390/nu18040608 (PMC12943266; doi:10.3390/nu18040608)
Supplement: Supplementary file 1 [file nutrients-18-00608-s001.zip › Supplementary Material 3 List of studies.pdf]

Lp.	Title	doi	Year	Country	Species	Sample size (N)	Groups
1	Acetobacter pasteurianus BP2201 alleviates alcohol-induced hepatic and neuro-toxicity and modulate gut microbiota in mice	https://doi.org/10.1111/1751-7915.14303	2023	China	Female C57BL/6J mice	40	Short-term cognitive dysfunction experiment: ConS (n=8), EtOHS (n=8), ProbioticsS (n=8). Long-term liver injury experiment: ConL (n=6), EtOHL (n=4), ProbioticL (n=6).
2	Akkermansia muciniphila Ameliorates Alcoholic Liver Disease in Experimental Mice by Regulating Serum Metabolism and Improving Gut Dysbiosis	10.3390/metabo13101057	2023	China	Female C57BL/6J mice	32	Con+PBS (pair-fed control); EtOH+PBS (ethanol only); EtOH+Akk (ethanol + A. muciniphila); Con+Akk (control + A. muciniphila).
3	Alcoholic Liver Disease: A Mouse Model Reveals Protection by Lactobacillus fermentum	10.1038/ctg.2015.66	2016	Italy	Female BALB/cAnNHsd mice	48	Con (n=13); 4EtOH (n=5); 8EtOH (n=5); 12EtOH (n=5); 4EtOH-P (EtOH + probiotic, n=5); 8EtOH-P (n=5); 12EtOH-P (n=5); 8EtOH-4P (EtOH 8 weeks + probiotic 4 weeks, n=5).
4	Antioxidative and Anti-Inflammatory Effects of Lactobacillus plantarum ZS62 on Alcohol-Induced Subacute Hepatic Damage	https://doi.org/10.1155/2021/7337988	2021	China	Male Kunming mice	40	Con (control); EtOH (alcohol only); EtOH + L. delbrueckii subsp. bulgaricus (LDSB); EtOH + L. plantarum ZS62.
5	Bacillus coagulans regulates gut microbiota and ameliorates the alcoholic-associated liver disease in mice	10.3389/fmicb.2024.1337185	2024	China	C57BL/6 male mice	45	Con (control); EtOH (ethanol); EtOH + B. coagulans.
6	Bifidobacterium breve ATCC15700 pretreatment prevents alcoholic liver disease through modulating gut microbiota in mice exposed to chronic alcohol intake	https://doi.org/10.1016/j.jff.2020.104045	2020	China	Male C57BL/6J mice	40	Con (pair-fed); EtOH-fed; B. breve + EtOH; B. breve only.
7	Dietary Bacillus subtilis supplementation alleviates alcohol-induced liver injury by maintaining intestinal integrity and gut microbiota homeostasis in mice	10.3892/etm.2021.10747	2021	China	Male C57BL/6J mice	40	Con; EtOH; B. subtilis lactis; EtOH + B. subtilis.
8	Efficiency of double layered microencapsulated probiotic to modulate proinflammatory molecular markers for the management of alcoholic liver disease	https://doi.org/10.1155/2014/715130	2014	India	Female Wistar rats	48	Con; EtOH; EtOH + free L. plantarum; EtOH + encapsulated L. plantarum; free L. plantarum; encapsulated L. plantarum.

9	Hepatic and Fecal Metabolomic Analysis of the Effects of Lactobacillus rhamnosus GG on Alcoholic Fatty Liver Disease in Mice	https://doi.org/10.1021/pr501121c	2015	USA	Male C57BL/6 mice	22	Con (pair-fed, no LGGs, n=5); Con + LGG (pair-fed + LGGs, n=7); EtOH (EtOH-fed, no LGGs, n=4); EtOH + LGG (EtOH-fed + LGGs)
10	Hepatoprotective effects of Lactococcus chungangensis CAU 1447 in alcoholic liver disease	10.3168/jds.2019-16891	2019	South Korea	Sprague-Dawley rats	30	Negative control (PBS); Positive control (EtOH only); Drug control (EtOH + silymarin); EtOH + L. chungangensis CAU 1447.
11	Komagataeibacter hansenii CGMCC 3917 alleviates alcohol-induced liver injury by regulating fatty acid metabolism and intestinal microbiota diversity in mice	https://doi.org/10.1039/c9fo02040c	2020	China	Male Kunming mice	40	Con (n=10); EtOH-treated (n=10); EtOH + low-dose Komagataeibacter hansenii CGMCC 3917 (2×10^6 CFU/mL, n=10); EtOH + high-dose Komagataeibacter hansenii CGMCC 3917 (2×10^8 CFU/mL, n=10
12	Lacticaseibacillus rhamnosus CCFM1060 Modulates gut microbiota and intestinal barrier Function: Alcoholic liver disease Mitigation through Nrf2/HO-1 and NF-κB Pathways	https://doi.org/10.1016/j.jff.2024.106516	2024	China	Male C57BL/6J mice	Not explicitly reported.	Con; EtOH; viable Lacticaseibacillus rhamnosus; heat-killed L. rhamnosus; comparative probiotic (L. rhamnosus GG)
13	Lacticaseibacillus rhamnosus LRa05 alleviated liver injury in mice with alcoholic fatty liver disease by improving intestinal permeability and balancing gut microbiota	https://doi.org/10.1163/18762891-bja00022	2024	China	Male C57BL/6J mice	30	Con (control); EtOH (ALD); L. rhamnosus LRa05 intervention (LRa05)
14	Lactobacillus GG treatment ameliorates alcohol-induced intestinal oxidative stress, gut leakiness, and liver injury in a rat model of alcoholic steatohepatitis	https://doi.org/10.1016/j.alcohol.2008.12.009	2009	USA	Male Sprague-Dawley rats	28	EtOH + vehicle (ALC-V, n=11); EtOH + L. rhamnosus GG (ALC-LGG, n=9); dextrose control (CON, n=5); dextrose + L. rhamnosus GG (CON-LGG, n=3)
15	Lactobacillus helveticus attenuates alcoholic liver injury via regulation of gut microecology in mice	https://doi.org/10.1111/1751-7915.70016	2023	China	C57BL/6 mice	Not explicitly reported.	Con (control diet + vehicle); EtOH (EtOH diet + vehicle); EtOH + L. helveticus (LH); EtOH + L. casei (LC); EtOH + L. paracasei (LP); EtOH + L. sakei (LS); EtOH + L. delbrueckii (LD)
16	Lactobacillus plantarum J26 alleviates alcohol-induced oxidative liver injury by regulating the Nrf2 signaling pathway	https://doi.org/10.26599/FSHW.2022.9250172	2024	China	Male C57BL/6J mice	50	Con (Normal control); EtOH (Alcohol model); LLP (low-dose L. plantarum J26); MLP (medium-dose L. plantarum J26);

							HLP (high-dose L. plantarum J26)
17	Lactobacillus plantarum J26 Alleviating Alcohol-Induced Liver Inflammation by Maintaining the Intestinal Barrier and Regulating MAPK Signaling Pathways	https://doi.org/10.3390/nu15010190	2022	China	C57BL/6J mice	50	Con (normal control); EtOH (Alcohol model); LLP (L. plantarum J26, 10^7 CFU/mL); MLP (L. plantarum J26, 10^8 CFU/mL); HLP (L. plantarum J26, 10^9 CFU/mL)
18	Lactobacillus rhamnosus CCFM1107 treatment ameliorates alcohol-induced liver injury in a mouse model of chronic alcohol feeding	https://doi.org/10.1007/s12275-015-5239-5	2015	China	Male Kunming mice	60	Skim milk; EtOH + skim milk; EtOH + L. rhamnosus CCFM1107; E tOH + Hu-Gan-Pian (HGP); EtOH + L. rhamnosus GG (LGG); EtOH + L. plantarum CCFM1112
19	Lactobacillus rhamnosus GG reduces hepatic TNFα production and inflammation in chronic alcohol-induced liver injury	https://doi.org/10.1016/j.jnutbio.2013.02.001	2013	USA	Male C57BL/6N mice	Not explicitly reported.	Con (pair-fed control); EtOH (EtOH-fed); EtOH + L. rhamnosus GG (AF+LGG)
20	Lactobacillus rhamnosus GG treatment potentiates intestinal hypoxia-inducible factor, promotes intestinal integrity and ameliorates alcohol-induced liver injury	10.1016/j.ajpath.2011.08.039	2011	USA	Male C57BL/6N mice	Not explicitly reported.	Con (pair-fed); EtOH (EtOH-fed); EtOH + LGG; normal chow (N)
21	Lactobacillus rhamnosus Granules Dose-Dependently Balance Intestinal Microbiome Disorders and Ameliorate Chronic Alcohol-Induced Liver Injury	10.1089/jmf.2018.4357	2020	China	Male C57BL/6N mice	64	Con (pair-fed, n=7); EtOH (EtOH-fed, n=12); EtOH+LGGL (low dose, n=15); EtOH+LGGM (medium dose, n=15); EtOH+LGGH (high dose, n=15)
22	Lactobacillus rhamnosus NKU FL1-8 Isolated from Infant Feces Ameliorates the Alcoholic Liver Damage by Regulating the Gut Microbiota and Intestinal Barrier in C57BL/6J Mice	10.3390/nu16132139	2024	China	Male C57BL/6J mice	24	Con (control, n=8); EtOH (EtOH model, n=8); L. rhamnosus NKU FL1-8 (FL1-8, n=8)
23	Levilactobacillus brevis MG5311 Alleviates Ethanol-Induced Liver Injury by Suppressing Hepatic Oxidative Stress in C57BL/6 Mice	https://doi.org/10.3390/microorganisms10122488	2022	Republic of Korea	Male C57BL/6 mice	18	Con (normal diet, n=6); EtOH diet (n=6); EtOH + L. brevis MG5311 (MG5311, n=6)
24	Liver Metabolomics Reveals the Effect of Lactobacillus reuteri on Alcoholic Liver Disease	10.3389/fphys.2020.595382	2020	China	Male C57BL/6 mice	18	Con (maltose dextrin instead of EtOH); EtOH-fed; EtOH + L. reuteri
25	Metagenomic analyses of alcohol induced pathogenic alterations in the intestinal microbiome and the effect of Lactobacillus rhamnosus GG treatment	https://doi.org/10.1371/journal.pone.0053028	2013	USA	Male C57BL/6N mice	20	Con (pair-fed, n=8); EtOH (EtOH-fed, n=8); EtOH + LGG (n=4)

26	New strain of Pediococcus pentosaceus alleviates ethanol-induced liver injury by modulating the gut microbiota and short-chain fatty acid metabolism	https://doi.org/10.3748/wjg.v26.i40.6224	2020	China	C57BL/6 mice	28	Con (pair-fed + PBS, n=8); EtOH (ethanol-fed + PBS, n=10); P. pentosaceus (EtOH-fed + P. pentosaceus, n=10)
27	Protective effects of Lactobacillus plantarum against chronic alcohol-induced liver injury in the murine model	https://doi.org/10.1007/s00253-019-10122-8	2019	China	Adult male C57BL/6J mice	48	Con (control, saline); Plac (placebo, isocaloric lactose); H (high-dose L. plantarum CMU995, 1×10^{10} CFU/mouse/day); M (medium-dose, not specified); L (low-dose, not specified); S (positive control, silymarin 25 mg/kg/day)
28	Protective effects of probiotics on acute alcohol-induced liver injury in mice through alcohol metabolizing enzymes activation and hepatic TNF-α response reduction	https://doi.org/10.1016/j.jff.2019.05.018	2019	China	Male C57BL/6 mice	96	Con; EtOH; YRT3115 (B. bifidum YRT3115); KV9 (B. animalis subsp. lactis KV9)
29	Pyrroloquinoline quinone-secreting probiotic Escherichia coli Nissle 1917 ameliorates ethanol-induced oxidative damage and hyperlipidemia in rats	https://doi.org/10.1111/acer.12456	2014	India	Male Charles Foster albino rats,	48	Chronic study: Con; EtOH; vitamin C; PQQ (extract); E. coli Nissle 1917 (EcN): EcN-2; EcN-3; EcN-4
30	Recovery of Bacteroides thetaiotaomicron ameliorates hepatic steatosis in experimental alcohol-related liver disease	https://doi.org/10.1080/19490976.2022.2089006	2022	Italy	C57BL/6 mice	Not explicitly reported.	Con (Pair-fed vehicle); Bt (Pair-fed B. thetaiotaomicron); EtOH vehicle; EtOH + Bt.<br><br>(Initial comparisons: pair-fed n=5–6, EtOH n=6–10; intervention phase: pair-fed n=3–6, EtOH n=4–10.)
31	Study on the Regulatory Mechanism of Niacin Combined with B. animalis F1-7 in Alleviating Alcoholic Fatty Liver Disease by Up-Regulating GPR109A	https://doi.org/10.3390/nu16234170	2024	China	male C57BL/6N mice	75	Con; EtOH model (AFLD); probiotic F1-7; probiotic + niacin; niacin only
32	The amelioration of alcohol-induced liverand intestinal barrier injury by Lactobacillus rhamnosus Gorbach-Goldin (LGG) is dependent on Interleukin 22 (IL-22) expression	https://doi.org/10.1080/21655979.2022.2070998	2022	China	male C57BL/6	Not explicitly reported.	Con (control diet); EtOH diet; EtOH + Lactobacillus rhamnosus GG; Lactobacillus rhamnosus GG
33	The effect of Lactobacillus rhamnosus B10 on alcoholic liver injury and intestinal microbiota in alcohol-induced mice model	https://doi.org/10.1111/jfbc.14372	2022	China	Male C57BL/6N	40	Con (Lieber–DeCarli control diet); EtOH-fed (5% v/v, n=15); EtOH + L. rhamnosus B10 (AF+LC, n=15)

34	Therapeutic Potential of Lactiplantibacillus plantarum FB091 in Alleviating Alcohol-Induced Liver Disease through Gut-Liver Axis	https://doi.org/10.4014/jmb.2407.07051	2024	Korea	Male C57BL/6J mice	12	Con (control); EtOH (EtOH-fed); EtOH + L. plantarum FB091; EtOH + L. plantarum ATCC 8014
35	Symbiotic combination of Akkermansia muciniphila and inosine alleviates alcohol-induced liver injury by modulating gut dysbiosis and immune responses	10.3389/fmicb.2024.1355225	2024	China	male C57BL/6 mice	50	Con (control diet); EtOH (EtOH fed); AKK (A. muciniphila); I (inosine); AKK + I (A. muciniphila + inosine)
36	Protective Effect of Enterococcus faecium Against Alcohol-Induced Acute Liver Injury Via Extracellular Vesicles in Rats	http://dx.doi.org/10.1089/fpd.2025.0005	2025	China	Sprague Dawley (SD) rats	24	Normal Control (PBS) Ethanol Model (EtOH) Enterococcus faecium (Efm)
